# MRI With Intratympanic Gadolinium: Comparison Between Otoneurological and Radiological Investigation in Menière's Disease

**DOI:** 10.3389/fsurg.2021.672284

**Published:** 2021-06-04

**Authors:** Giampiero Neri, Armando Tartaro, Letizia Neri

**Affiliations:** ^1^Neurosciences, Imaging and Clinical Sciences Department, Gabriele d'Annunzio University, Chieti, Italy; ^2^Medical, Oral and Biotechnologies Sciences Department, Gabriele d'Annunzio University, Chieti, Italy

**Keywords:** magnetic resonance imaging, intratympanic gadolinium, vestibular evoked myogenic potential, head impulse test, pure tone audiometry, caloric bithermal test, posturography, Menière's disease

## Abstract

**Objectives/hypothesis:** To compare findings obtained using both magnetic resonance imaging plus intratympanic gadolinium and audiovestibular testing for Menière's disease.

**Study design:** Retrospective cohort study.

**Methods:** Patients with definite unilateral Menière's disease (*n* = 35) diagnosed according to 2015 Barany Criteria were included. Three-dimensional real inversion recovery (3D-real-IR) MRI was executed 24 h after intratympanic gadolinium injection to assess the presence and degree of endolymphatic hydrops. Pure tone audiometry, bithermal caloric test, head impulse test, ocular, and cervical VEMPs using air-conducted sound were performed to evaluate the level of hearing and vestibular loss. The results were compared to verify precision of the method in providing correct diagnoses.

**Results:** Different degrees of endolymphatic hydrops were observed in the MRI of the cochlea and vestibule in the affected ears of Menière's disease patients, even though it was impossible to radiologically distinguish the two otolithic structures separately. The correlation between the degree of linked alterations between instrumental and MRI testing was statistically significant. In particular, an 83% correspondence with audiometry, a 63% correspondence for cVEMPs and 60% correspondence for cVEMPs were seen. While for HIT the accordance was 70 and 80% for caloric bithermal test.

**Conclusions:** MRI using intratympanic gadolinium as a contrast medium has proved to be a reliable and harmless method, even though there is an objective difficulty in disclosing macular structures. The study revealed that there is no complete agreement between instrumental values and MRI due to the definition of the image and fluctuation of symptoms. The present work highlights the greater (but not absolute) sensitivity of otoneurological tests while MRI, although not yet essential for diagnosis, is certainly important for understanding the disease and its pathogenic mechanisms.

## Introduction

Menière's disease (MD) also called “idiopathic syndrome of endolymphatic hydrops” (EH) (1) and named after Prosper Menière (2) is a chronic inner ear disease whose symptoms are vertigo with hearing loss, tinnitus and aural pressure. Its official diagnosis is based on guideline symptoms (3) and on pure tone audiogram. Recent vestibular tests can assess dysfunctions of the cochlea, otolith organs and semicircular canals. In 2007 Nakashima (4, 5) using MRI imaging with intratympanic gadolinium demonstrated endolymphatic hydrops (6) in Menière's ears. Since then, this technique has been used to evaluate hydrops, the size and shape of endolymphatic spaces, and the severity and evolution of MD (7). Not only is endolymphatic hydrops on MRI an indicator of MD, but also of other disorders (8–10). Variability of diagnosis is reflected in epidemiological studies (11, 12) including patients in which the full range of symptoms is not present (13). A comparison between audiovestibular symptoms and tests and MRI inner ear imaging might improve MD diagnosis. In the present study the results obtained through MRI inner ear imaging were compared with audiological findings in patients with a clinical diagnosis of MD according to the 2015 Barany Criteria (3).

## Materials and Methods

This retrospective clinical study was conducted in collaboration with Biomedics Advanced Technologies Institute and Ear Nose and Throat Department of Chieti-Pescara University Hospital. The patients had a definite unilateral MD and were subjected to MRI 3T and intratympanic injection of gadolinium (Gd) (1 mmol/ml) diluted 1:7 in saline in the affected ear. The study protocol was approved by the local ethics committee. All patients signed informed consent and the study was conducted in accordance with the Declaration of Helsinki of 1975 revised in 2013.

Thirty-five (14) volunteer adult patients (19 females and 16 males) aged between 38 and 72 years (mean age 52.83) who arrived at the Hospital as out-patients for unilateral definite MD according to the 2015 Barany Criteria (3) were included in the study. Patients with bilateral MD, patients previously subjected to ear surgery, patients subjected to intratympanic treatment with gentamicin, patients with vestibular migraine, patients suffering from intracranial neoplasms, and patients in whom MRI was contraindicated were excluded from the study. No patients included in the study had acute phase MD. Three-dimensional real inversion recovery (3D-real-IR) MRI was executed 24 h after intratympanic gadolinium injection to assess the presence and degree of endolymphatic hydrops (EH). Fisher *T*-test was used for statistical evaluation.

Magnetic Resonance Imaging (MRI) was performed and the contrast medium (0.4/0.5 ml) was injected intratympanically using a 1 ml syringe with a 23G needle and a dilution of 1 cc of Gd in 7cc of saline with a 1:7 ratio. After the injection, the patient remained supine and with the head turned 45° toward the affected side, for about 30 min. Three dimensional real inversion recovery (3D-real-IR) MRI was conducted 24 h after the intratympanic gadolinium injection to assess the presence and degree of endolymphatic hydrops (EH). All tests were evaluated according to Nakashima grading (15) which is based on hydrops volume ratios in FLAIR/T2 (G) from 0 to 1 and expressed in 3 degrees of severity (Table 1). Since the normal ratio range of the endolymphatic area over the vestibular fluid space (sum of the endolymphatic and perilymphatic areas) was 33%, any ratio increases over 33 % were considered positive.

**Table 1:** Hydrops visualization grading, represented by the ratio between the volumes of the contrast agent in the Flair 3D and TSE and T2 sequences.

**Grading**	**Ratio**	**Results**
0	0–0.33	No hydrops
1	0.33–0.66	Middle gravity hydrops
2	0.66–1	Severe hydrops

Pure tone audiometry (PA) was bone and air conducted using Amplaid audiometer. The difference between air and bone hearing thresholds (cochlear reserve) was evaluated using 250-500-1,000 frequencies, and the results were considered as indicative of MD in patients with cochlear reserve >10 Db on at least one of these frequencies.

Bithermal caloric test (BCT) was performed using the Fitzgerald Hallpike method with water at 30 and 44°C. The angular velocity of the slow phase was calculated using the Jongkees formula. Patients with a labyrinthine preponderance >20% were considered positive for labyrinth damage.

Digital Video head impulse test (vHIT) was performed on the lateral canal using only ICS Impulse vHIT GN Otometrics, (Denmark) and the VOR gains were measured taking into account mean eye velocity divided by mean head velocity during a 40 ms window centered at the time of peak head acceleration. Abnormal VOR gain was considered in the presence of corrective saccades after the head movement. The decreased gain for the horizontal canal was calculated outside the mean 2 SDs compared to normal controls (<0.70 or> 0.999) (13).

Ocular (oVEMP) and Cervical VEMPs (cVEMP) were tested using air-conducted sound and altered VEMP was considered when the amplitude of the affected side was lower or absent as compared to the opposite. Two consecutive runs were averaged to provide a final response. A Stabilometry test was performed to evaluate the level of hearing and vestibular loss.

Posturography (PG) was carried out on a baropodometric platform (Phisionorm NBP Computerized Posturographic System) a few hours before and about 20–24 h after the intratympanic injection of the contrast medium. The test was conducted with both opened and closed eyes using a foam cushion between the feet and the platform, which could alter somatosensory and proprioceptive information, and respecting an interval of 30 s between one measurement and the next.

## Results

**Figure 1:**
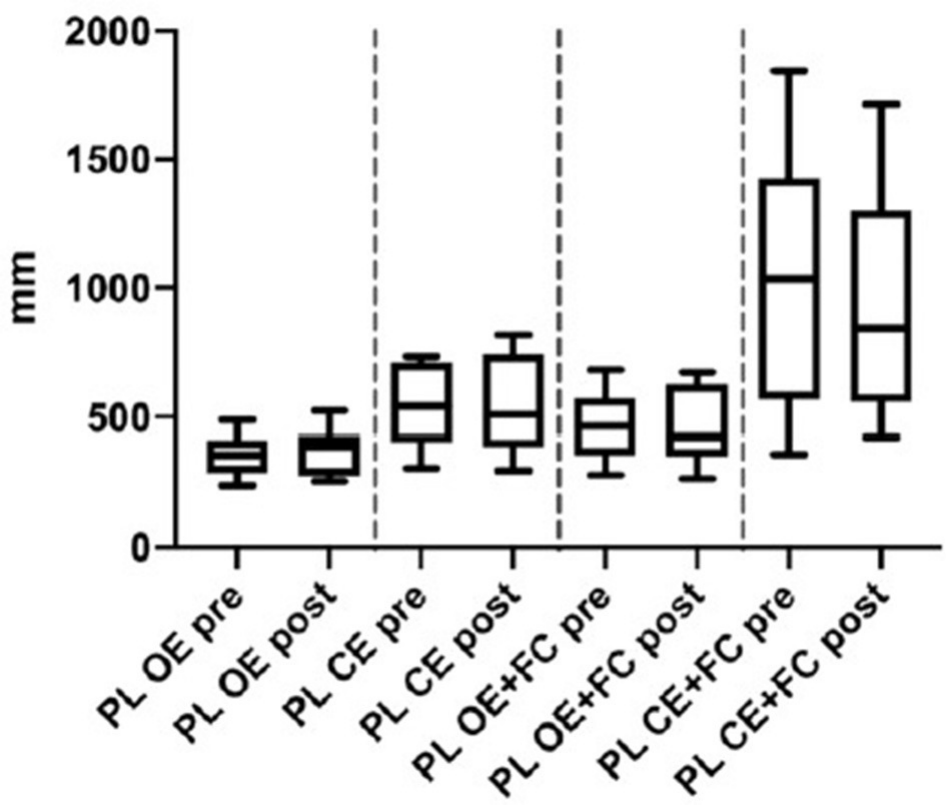
Posturography: Path length of Menièr's patients before (pre) and 24 h- after (post) the intratympanic injection. OE, Open Eyes; CS, Closed Eyes; PC, Foam cushion under feet. Data we presented as boxes and whiskers (Tukey method).

In all 35 patients no hearing loss, vestibular complications or ear drum perforations after gadolinium injection could be evidenced. In four patients who had all reported to have suffered from otitis in childhood, the contrast medium could not reach the labyrinth due to lack of diffusion through the round window. One of the patients (3%) refused to perform the second MRI. These five patients were excluded from the study. In the remaining 30 patients in the study group, distension of the endolymphatic spaces was observed in 22 (73%) while the labyrinth showed no signs of hydrops in the remaining eight (27%). No patients had their cochlear aqueduct lightened by gadolinium, while in all 30 patients in which the intratympanic infiltration lightened the labyrinth, gadolinium in the internal auditory canal (Figure 1) could be seen after 24 h.

The PA showed the presence of a constant transmission component on the 250-500-1000 frequencies that was inversely proportional to the severity of the sensorineural hearing loss (Table 2), with an average cochlear reserve of 22.3 dB at 250 Hz, of 12.3 Db at 500 Hz, and 5.2 Hz at 1,000 Hz. When comparing the results obtained with MRI and audiometric testing (Table 3) a significant correlation could be evidenced in 25 out of 30 patients (83.3%) (*p* = 0.002).

**Table 2:** Cochlear reserve in decibels on the frequencies 250-500-1000 Hz of each patient and calculations of the average cochlear reserve.

**Hz**	**250**	**500**	**1,000**
Decibel	−15	−5	−5
	−10	−5	−5
	−40	−20	−5
	−30	−20	−10
	−15	−5	−5
	−15	−5	−5
	−35	−25	−10
	−25	−20	−5
	−45	−30	−10
	−25	−10	−5
	−25	−20	−5
	35	20	10
	−30	0	0
	−35	−10	−5
	−30	0	0
	−40	−20	−5
	−35	−15	−5
	−10	−5	−5
	−25	−20	−5
	−35	−25	−10
	−40	−20	−5
	−25	−20	−5
	−30	−20	−10
	−30	−25	−10
	−35	−10	−5
	−25	−10	−5
	−5	−5	−5
	−10	−5	−5
	−5	−5	−5
	−20	−10	−5
Average cochlear reserve	**−22.3**	**−12.3**	**−5.2**

**Table 3:** Ratio between positive and negative patients on MRI and patients with cochlear reserve >10 dB on the frequencies 250-500-1000 Hz (*p* = 0.002) sensibility 86%.

**Audiometry—MRI correspondence**		
	**MRI+**	**MRI-**
Audiometry +	20	3
Audiometry −	2	5

Vestibular evoked potentials were tested using air-conducted sounds and showed radiological-electrophysiological concordance in 63% of the patients (19/30) with either absent or reduced saccular (cVEMPs) bioelectric signals and in 60% of the patients with utricular (VEMPs) bioelectric signals (Table 4), these data being not statistically significant (0.3–0.5). On the contrary, the MRI method used was not able to distinguish the saccule from the utricle separately.

**Table 4:** Ratio between positive and negative patients on MRI and patients with and subjects with absent or reduced evocability of VEMPs (*p*CVEMPs = 0.3, *p*OVEMPs = 0.5).

**VEMPs - MRI correspondence**		
	**RM** **+**	**RM -**
C-VEMPS +	15	4
C-VEMPS -	7	4
O-VEMPS +	16	5
O-VEMPS -	6	3

Video HIT based solely on the horizontal canal showed the presence of a significant (*p* = 000.3) correspondence in 70% of the MD patients (21/30 patients) (Table 5).

**Table 5:** Ratio between positive and negative patients on MRI and patients with corrective saccades after the head movement and decreased gain for the horizontal canal (*p* = 0.003).

**vHIT - RM correspondence**		
	**RM+**	**RM-**
HIT +	15	2
HIT -	7	6

The bithermal caloric test showed a significant correlation between damage and radiological labyrinthine hydrops positivity of 80% (24/30) (*p* = 0004), while in 20% of the patients (6/30 patients) labyrinthine hydrops could not be demonstrated radiologically, even with a positive caloric test (Table 6).

**Table 6:** Bithermal Caloric Test: Ratio between positive and negative patients on MRI and patients with a preponderance >20% (*p* = 0.004).

**Bithermal caloric test—RM**		
	**RM** **+**	**RM -**
BCT +	16	0
BCT -	6	8
Corrispondence	80%	
Discrepancy	20%	

In the computerized static posturography thes, the most important differences among path lengths (PL) were seen with closed eyes plus foam cushion under feet, and a significant improvement was observed 24 h after gadolinium injection (Figure 1) (*p* = 0.011, Cohen's *d* = 0.876, BF10 = 5.328) (data already reported elsewhere) (16).

**Figure 2:**
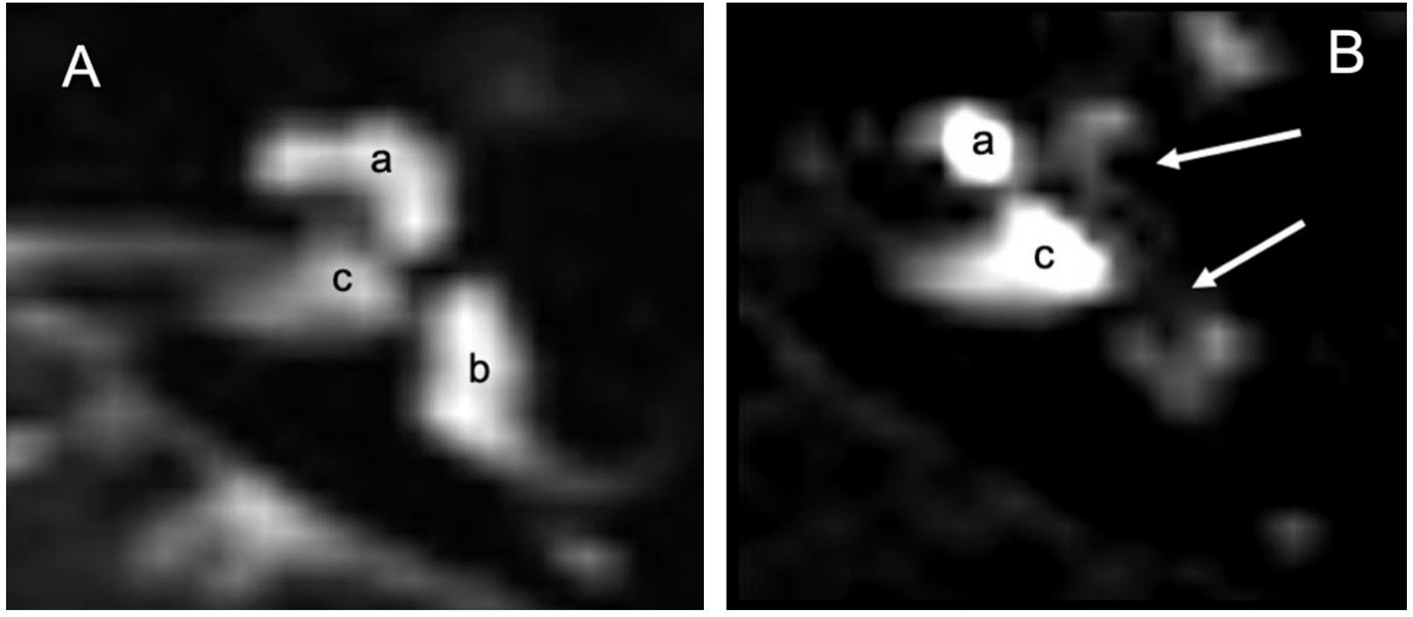
MRI 3 Tesla: **(A)** MRI in T2 without contrast means: labyrinthine fluids including peri and endolymphatic spaces are observed (a) cochlea, (b) vestibular spaces and CSL and internal auditory canal (c). **(B)** 24 h after infiltration with the Flair technique, the contrast shows absence of gadolinium in the apical gyrus of the cochlea and vestibule (white arrows). Gd illuminates part of the cochlea (a) and the internal auditory canal (c) which seems to be the main way of diffusion of gadolinium toward the liquor spaces.

Gadolinium MRI 3 Tesla did not cause any local or systemic adverse reactions. Thirty (17) of the 35 patients (86%) had a positive Gd response with contrast medium diffusion into the perilymph. Five (4) non-responders were excluded from the study. One radiologist graded the hydrops in different parts of the vestibule (Table 7). Contrast medium was observed in the internal auditory canal (Figure 2), demonstrating a communication between CSF and cochlear perilymph as described by Naganawa (18) and Kawai (19).

**Table 7:** Distribution of gadolinium in the perilymphatic spaces expressed in number of patients and percentage.

**Cochlea**	**Vestibular**	**Anterior**	**Posterior**	**Lateral**
	**spaces**	**SC**	**SC**	**SC**
21 (70%)	27 (90%)	13 (43%)	17 (56%)	13 (43%)

## Discussion

MD is the prototype of chronic recurrent diseases of the inner ear. Certain Meniere's disease diagnoses may be difficult despite the 2015 Barany Criteria, since they can be mimicked by other vestibular disorders. The caloric test has been the only available objective test and it has been based on the horizontal canal, and 3D-FLAIR MRI inner ear imaging and audiovestibular tests have allowed more precise diagnoses. More recently, the demonstration of inner ear hydrops by Naganawa and Nakashima (4) has granted to use MRI in MD and to disclose hydrops in migraine, trauma, sudden deafness, vestibular schwannoma, and semicircular canal dehiscence (20–22). However, doubts remain concerning the correlation between radiological and audio-vestibular findings. Pyykko et al. (9) have found a 53% sudden hearing loss in patients with EH and Katayama et al. (23) and Seoa et al. (24) have reported conflicting results as to whether cVEMPs and oVEMPs are concealed by hydrops; for caloric tests controversial findings have also been reported in literature (17, 25, 26).

Some authors have reported MD patients having a normal vHIT in the presence of a canal paresis on bithermal caloric testing. It has been hypothesized that hydropic distension allows endolymph recirculation with dissipation of its hydrostatic force and reduction of cupula displacement and nystagmus (27), and this hypothesis could have been confirmed by a direct observation of the vestibules in these patients. In the present study none of the methods, including MRI, achieved absolute diagnostic capacity. In particular, the bithermal caloric test showed a significant concordance between labyrinthine disease and radiological hydrops in 80% of the patients. The data in the present study are in line with the literature, and we and Dumas (28) have suggested that the discrepancy could be the result of either hydrops fluctuation that improves cochlear and vestibular function between attacks or of a mistaken diagnosis. The data of the present study are therefore in line with what emerges from literature, and indicate that caloric testing was able to evidence labyrinthine damage but not hydrops, as it was negative in 20–30% of the patients. Probably the reason for such discrepancy lies in the fluctuation of the hydrops in the patients in the present study during inter-critical periods, as in those by Dumas (28), which reduces the endolymphatic pressure thus improving both cochlear and vestibular function.

Evaluation of the horizontal canal using both caloric test and vHIT resulted in a reduction of agreement with MRI from 80 to 70%. The two methods stimulated the labyrinth with two different modalities, by 0.03 Hz frequencies in the caloric test, and by angular accelerations that could even reach 2,000–4,000°/s2 in the HIT (14) and this data confirm that in MD the horizontal semicircular canal maintains the ability to be stimulated on the low frequencies better than on the high ones. The most prominent feature of VOR was indeed adaptation (29). The labyrinth is able to change and improve against harmful events that might arise either slowly such as in aging, or rapidly such as in the case of lesions of the peripheral vestibular organ. In all cases, however, the labyrinthine asymmetry was reduced, but the response of the VOR to low frequencies and to low speed head movements recovered within a few weeks, while the responses to high frequency stimuli (such as HIT) were rarely complete and therefore an asymmetry to rapid head rotations persisted (30), which generated the saccadic movement toward the injured side, typical of the HIT (31).

In the present study, patients were subjected to audiometric tests, and the focus was on the evaluation of low frequencies (250-500-1000), neglecting sensorineural loss resulting from MD and hearing fluctuations as they have already been widely described in the literature and represent an essential part of commonly used diagnostic criteria. Instead, the focus was on the presence or absence of cochlear reserve, which is mainly determined by the pressure of the endolymph on the platen of the stirrup and which therefore was indicative of hydrops rather than just of hearing loss. The present data show that the cochlear reserve corresponded to the MRI endolymphatic hydrops in a significant percentage of patients (83%) and therefore the cochlear reserve represents the simplest and most immediate finding that indicated hydrops regardless of sensorineural hearing loss, with a sensitivity of 86%. The spinal vestibule reflex was also evaluated using VEMPs, which did not prove to be comparable with MRI in quantifying the damage. This seems to be a consequence of the wide variability of both cervical and ocular responses in VEMPs (32), which changed according to the stage of the disease, so that in the acute phase oVEMPs increased while the cVEMPs decreased in amplitude (17) and to the vestibular nervous branch involved. Indeed, it has been observed that damage to the superior vestibular nerve results in a normal response of the cervical VEMPs and in a reduced or absent response to the ocular VEMPs while the opposite occurs with damaged inferior vestibular nerve (33–35). Both these phenomena occur because in MD the ocular VEMPs have a lower response compared to the cervical VEMPs, and it has been hypothesized that the utricle is more associated with the auditory function at low frequencies as compared to the saccule. In the present work, the posturographic evaluations showed that vestibular function not only does not worsen, but it even improves (16) after intratympanic injection, and vestibular function was certainly not linked to the Gd administration. Instead, as for audiometry, vestibular function could be linked to the pressure changes in the middle ear, which are induced by the mass of injected fluid, exerting a micro-pressure on the oval window and so modifying the dynamics inside the labyrinth as already described in Therapy with Meniett by Odqvist (36) and Gates (37).

A final observation that emerged from the imaging analysis, concerns the diffusion of the contrast medium in the internal auditory canal (IAC) (Figure 2). The IAC is lined with dura mater and arachnoid and it is perforated at the bottom by the vascular-nervous bundle which includes the VII and VIII cranial nerves. In IAC therefore, a natural communication exists between the cerebrospinal fluid (CSF) and perilymph (38). The MRI representations of this fluid exchange confirmed how CSF pressure changes can be transmitted to the perilymph, and therefore to the cochlea. This contiguity determines CSF pressure increases, as in gusher syndrome during stapedotomy, and in CSF pressure reductions, as in patients undergoing peritoneal ventricular shunt. Such shunts result in a low perilymphatic pressure with relative endolymphatic hydrops (39) and in variations in otoemission due to a “vacuum” in the inner ear (40, 41). These data seem to show that the relation between CSF and perilymph could be another etiopathogenetic mechanism of MD.

In the present work, intratympanic injection of Gd and subsequent 3T RM was certainly the most revolutionary way of using contrast medium in the vestibology field, as it was able to visualize endolymphatic hydrops and to ascertain not only cochlear hydrops but also a hydropic extension to the remaining parts of the posterior vestibule. However, intratympanic MRI assessment of hydrops is time-consuming, expensive and fails in about 20% of patients. The use of intravenous contrast medium could be a valid alternative to intratympanic injection as it could avoid the contrast medium diffusion problems encountered in this study. It is therefore necessary to associate imaging with electrophysiological tests, which are cheaper and easier to perform.

In this study, MRI showed results that are similar to those that have been seen for a long time with caloric tests and audiometry, which have a greater sensitivity than more recent tests such as HIT, which however showed reduced but significantly similar findings to MRI. VEMPs, on the contrary, even though retaining all their validity, due to their wide variability could be correlated with the imaging testing which should therefore be seen as the best choice. Beyond method comparisons, it is indubitable that MRI allowed vision of the hydrops, and its added value was that it stimulated research and gave the opportunity to observe a “secret” compartment without producing cochlear damage or instability. This fact, impossible in the past opened new diagnostic, pathophysiological and therapeutic horizons. In past as well as in the present work, MRI allowed disclosure of new data, such as the possible role played by the perilymph and cerebrospinal fluid in the etiopathogenesis of MD, and MRI combined with instrumental and electrophysiological data guarantee MD monitoring over time and the effectiveness of the therapy.

## Data Availability Statement

The original contributions presented in the study are included in the article/supplementary material, further inquiries can be directed to the corresponding author/s.

## Ethics Statement

The studies involving human participants were reviewed and approved by University of Chieti. The patients/participants provided their written informed consent to participate in this study.

## Author Contributions

GN ideate the work write the manuscript and revised the manuscript. AT performed the radiological examinations, helped write the work, and translate the paper. LN recruited the patients and provide all clinical information elaborate the data helped write the work. All authors contributed to the article and approved the submitted version.

## Conflict of Interest

The authors declare that the research was conducted in the absence of any commercial or financial relationships that could be construed as a potential conflict of interest.
